# Ecology of predator-induced morphological defense traits in *Daphnia longispina* (Cladocera, Arthropoda)

**DOI:** 10.1007/s00442-019-04588-6

**Published:** 2020-01-16

**Authors:** Erik Sperfeld, Jens Petter Nilssen, Shelby Rinehart, Klaus Schwenk, Dag Olav Hessen

**Affiliations:** 1 Animal Ecology, Zoological Institute and Museum, University of Greifswald, Greifswald, Germany; 2 Centre for Ecological and Evolutionary Synthesis (CEES), Department of Biosciences, University of Oslo, Blindern, Oslo, Norway; 3 Müller-Sars Society for Free Basic Research, P.O. Box 5831, 0308 Oslo, Norway; 4 Section for Aquatic Biology and Toxicology (AQUA), Department of Biosciences, University of Oslo, Blindern, Oslo, Norway; 5 Department of Ecology, Evolution, and Behavior, The Hebrew University of Jerusalem, Jerusalem, Israel; 6 Molecular Ecology, Institute for Environmental Sciences, University Koblenz-Landau, Landau in der Pfalz, Germany

**Keywords:** Chemical cue, Inducible defense, Morphological trait, Neck spine, Predator–prey interaction

## Abstract

Inducible defenses against predators are widespread among plants and animals. For example, some *Daphnia* species form neckteeth against predatory larvae of the dipteran genus *Chaoborus*. Though thoroughly studied in *D. pulex*, knowledge about neckteeth in other *Daphnia* species is limited. The occurrence of this trait in the *D. longispina* species complex is only sporadically reported and the specific shape of neckteeth or the occurrence of other morphological defense traits is scarcely known in this widespread group. Here, we explored neckteeth occurrence in a large number of *D. longispina* populations across Scandinavia and studied neckteeth formation and other morphological defense traits on three *D. longispina* clones in the laboratory. In the study region, neckteeth on juvenile *D. longispina* s. str. were observed frequently in permanent ponds, but only when *Chaoborus* spp. larvae were present. In the laboratory experiments, all three *D. longispina* clones developed neckteeth (very similar to *D. pulex*) in response to *Chaoborus* kairomone exposure. The *D. longispina* clones also developed a longer tail spine, wider body, and larger neckteeth pedestal in response to predation threat—likely as a defense against the gape-limited predator. The intensity of neckteeth expression also depended on the clone studied and the concentration of *Chaoborus* kairomone. Our results demonstrate that neckteeth on *D. longispina* can be common in nature and that *D. longispina* can also induce other morphological defenses against predators. The similarity of neckteeth in *D. longispina* and *D. pulex* imposes yet unresolved questions on the evolutionary origin in these distantly related *Daphnia* groups.

**Electronic supplementary material:**

The online version of this article (10.1007/s00442-019-04588-6) contains supplementary material, which is available to authorized users.

## Introduction

Induced anti-predator defenses are widespread among plants and animals and cover a wide range of chemical, behavioral, and morphological traits (Tollrian and Harvell 1999). Among invertebrates, the crustacean zooplankton *Daphnia* has become a widely used model organism for many reasons (Lampert 2011; Altshuler et al. 2011), not the least in the context of inducible defenses (e.g. Weiss et al. 2012). *Daphnia* spp. are widespread across temperate water bodies, and often constitute important prey for fish and invertebrate predators. Various species of *Daphnia* possess diverse anti-predator responses. For example, *Daphnia* exposed to fish predation commonly exhibit vertical migration behavior or changes in life history traits; while *Daphnia* exposed to invertebrate predators exhibit species-specific responses such as conspicuous helmets, spines, crests or neckteeth (Brehm 1909; Tollrian and Dodson 1999; Lass and Spaak 2003; Laforsch and Tollrian 2004; Weiss et al. 2012; Riessen and Gilbert 2019).

Neckteeth, also referred to as neck spines or Nackenzähne, are small extensions from the dorsal head margin. They are induced typically in early developmental stages as a specific defense against the predatory larvae of the “phantom midge”, *Chaoborus* spp., in several *Daphnia* species (Juračka et al. 2011; Riessen and Gilbert 2019). In particular, *D. pulex* has been used as a model system to study the causes, effects, and consequences of neckteeth induction (e.g. Krueger and Dodson 1981; Tollrian 1993; Tollrian and Dodson 1999; Hammill et al. 2008; Riessen and Trevett-Smith 2009). Neckteeth in juvenile *D. pulex* are induced by chemical cues (kairomones) released from actively feeding *Chaoborus* larvae and serve to protect individuals against predation (Krueger and Dodson 1981; Tollrian and Dodson 1999; Riessen and Trevett-Smith 2009). Specifically, the *Chaoborus* kairomone has to be present during the late phase of the embryonal development in the brood pouch of *D. pulex* mothers to induce neckteeth in juvenile offspring (Krueger and Dodson 1981; Imai et al. 2009; Weiss et al. 2016). The number of expressed neckteeth (typically 1–6 in *D. pulex*) can depend on *Chaoborus* kairomone concentration (Tollrian 1993; Hammill et al. 2008). Neckteeth formation is often accompanied by the development of a protrusion in the dorsal head region, at the basis of the neckteeth, called ‘neck-keel’ or ‘pedestal’, depending on magnitude. The occurrence and plasticity of the neck-keel or pedestal also depend on the strength of kairomone exposure (Tollrian 1993).

Neckteeth in *Daphnia* spp. occur in various forms and shapes, from single teeth to multiple teeth in rows or a rosette (“crowns”, Juračka et al. 2011), and are often accompanied by other defensive traits like elongated tail spines, increased body width, or more hidden defenses such as increased carapace thickness, strength, and stiffness (Laforsch et al. 2004; Riessen et al. 2012; Rabus et al. 2013; Kruppert et al. 2017; Riessen and Gilbert 2019). The induction of these traits in multiple species and species complexes within the genus *Daphnia* poses several interesting questions related to ecological–evolutionary interactions. On the one hand, neckteeth development may have evolved multiple times independently in different *Daphnia* species or species complexes (Colbourne et al. 1997; Kotov et al. 2006). This could be attributed to the dynamic and “ecoresponsive” genome of *Daphnia* (Colbourne et al. 2011). For example, some *Daphnia* groups show a complex history of multiple hybridization events and patterns of introgression as well as recent genetic adaptations, implying an active phase of speciation (Petrusek et al. 2008). On the other hand, the growing evidence of neckteeth occurrence in different, not closely related, *Daphnia* lineages may suggest a homologous origin, whereby neckteeth expression was only retained in taxa exposed to strong selection by *Chaoborus* spp. predation (Juračka et al. 2011).

Neckteeth formation is most studied within the *D. pulex* complex (see above), but is also described for other species or species complexes (e.g. Boronat and Miracle 1997; Lüning-Krizan 1997; Sell 2000; Benzie 2005; Kotov et al. 2006; Riessen and Trevett-Smith 2009; Juračka et al. 2010, 2011; Riessen and Gilbert 2019). Neckteeth (single, multiple, and rosettes) have also been reported in both pond and lake populations of the *D. longispina* complex (Juračka et al. 2011), covering formerly named *D. rosea* (Sell 2000, 2006), which is now assigned to the *D. longispina* group (Petrusek et al. 2008). The *D. longispina* complex, which has recently gone through major systematic revisions (Nilssen et al. 2007; Petrusek et al. 2008), is of particular interest because it can show similar neckteeth expression to *D. pulex*. This is surprising, given the assumption that *D. longispina* and *D. pulex* are reproductively isolated (neither hybridization nor introgression has been observed).

Not much is known about the general occurrence of neckteeth across *D. longispina* populations from different habitats. This may be due to the fact that neckteeth mostly occur in early developmental stages (typically within the first three instars), whilst taxonomy is generally based on adult females. *D. longsipina* is also more difficult to maintain in culture than larger *Daphnia* species such as *D. magna* or *D. pulex*, and thus is used less in laboratory experiments. This may explain why no thorough analysis of neckteeth development has been performed for this group and compared with patterns reported for *D. pulex*. Additionally, the various morphological responses that can accompany neckteeth induction (e.g. longer tail spines, wider bodies, or pedestal development) have not rigorously been tested in *D. longispina*. To fill these knowledge gaps, we first explored the occurrence of neckteeth in *D. longispina* in the field by screening many populations from a wide range of localities and habitats that differed in the presence of *Chaoborus* spp. predators. Next, we studied neckteeth induction across several juvenile stages in experiments using *D. longispina* clones originating from three geographically distant populations. Finally, we performed rigorous experimental testing of other morphological defense traits by accounting for allometric variation.

## Methods

### Field observations and historical records

As part of a continuous sampling of the biogeography of freshwater cladocerans and copepods in Fennoscandia and Denmark, the occurrence of neckteeth in *Daphnia* spp. has been recorded. The habitats investigated were sphagnum and grass wetlands, temporary and permanent ponds, rock-pools, and diverse lakes (e.g., small forest lakes and large inland lakes such as Vänern and Mälaren in Sweden). Around 800 locations (most in Norway) were investigated from 2004 to 2018 (see supplementary Fig. S1). Zooplankton was sampled with a plankton net (45 or 90 μm) or volume samplers. Samples were taken from the pelagic or shore regions.

We screened historical drawings and studies for evidence of neckteeth occurrence in the *D. longispina* group. We consulted publications from Swammerdam before 1700 and more recent papers until ~ 1950. Several studies reported neckteeth occurrence, but mostly within the *D. pulex* group. We went through the whole G. O. Sars’ Archives in the National Library Manuscript Department, Oslo, Norway (amounting to a paper stack of about 6 m length). G.O. Sars’ drawings from the early 1860s mainly showed neckteeth on *D. pulex*, but also a few unpublished drawings included *D. longispina* (see below). Additionally, we went through all available North American and East Asian papers until ~ 1950 to screen for neckteeth observations in *D. dentifera*, a closely related sister species of *D. longispina* s. str. that is assumed to belong to the *D. longispina* complex.

### Clone collection

Three clones of *Daphnia longispina* s. str. (Müller 1776), as defined in a study on the Palaearctic *Daphnia longispina* group (Petrusek et al. 2008), were collected at different, and geographically distant, locations in southern Norway (Fig. S1).

The clone Pond5–16 was collected on 16th June 2016 from a very small rock pool (~ 1 × 2 m) on an island in south-eastern Norway (~ GPS: 59.098405, 11.198153). The water of this rock pool was brownish (probably due to humic substances) and the small water body often dries out and reoccurs during summer in response to rainfall regimes. This locality has been sampled several times and *Chaoborus* spp. larvae have never been observed; however, Corixidae were often observed in small numbers. *D. magna* and *D. pulex* were found in this rock pool.

The clone AF-16 was collected on 14th August 2016 from a small artificial reservoir (10 × 15 m, max. depth ~ 0.4 m) in the old, inner city complex of Oslo, eastern Norway (GPS: 59.906224, 10.737012). The water was clear and there were no *Chaoborus* spp. larvae present at the time of sampling, but several Corixidae and some Notonectidae co-occurred. The reservoir is regularly drained for the winter season.

The clone GINA-17 was sampled on 17th September 2017 in a city pond of Haugesund, western Norway (~ GPS: 59.406704, 5.289214). This is a permanent pond of medium size (~ 25 × 35 m, max. depth 0.6 m) with a brownish water color. Both *Chaoborus* spp. larvae and Corixidae were present at the time of sampling. Early juvenile instars of *D. longispina* were dominated by males, and many of the juvenile males carried neckteeth with 1–3 spines (mostly 2–3 spines), whereas few of the rare juvenile females carried neckteeth (mostly 2 spines).

### Species delimitation of *Daphnia* clones

Besides morphological determination (using a microscope), the collected clones Pond5–16 and GINA-17 were subjected to genetic analyses using mitochondrial 12S rDNA and 10 microsatellite loci. The clone AF-16 could not be analyzed genetically as the culture ceased unexpectedly shortly after the experiments. DNA preparation using proteinase K digestion, amplification of mitochondrial and nuclear loci (DaB17/17, Dgm105, Dgm109, Dgm112, Dp196NB, Dp281NB, Dp519, SwiD6, SwiD14, SwiD18), and data analyses were conducted as described in previous studies (Schwenk et al. 1998; Petrusek et al. 2008; Thielsch et al. 2012). Mitochondrial DNA sequences were compared to reference sequences of species belonging to the *D. longispina* complex and nuclear multi locus genotypes were compared to a reference data set of 312 individuals belonging to either *D. galeata*, *D. cucullata* or *D. longispina* (Thielsch et al. 2017) using a model-based assignment test implemented in Structure 2.3.4 (Pritchard et al. 2000).

### General culture conditions

Daphnids were maintained in ADaM medium (Klüttgen et al. 1994) (modified using 0.05 times the recommended SeO_2_ concentration), fed the green algae *Chlamydomonas reinhardtii* (CC-1690 wild type mt+, Chlamydomonas Resource Center), and kept at 20 °C in temperature controlled rooms on a 16:8 light:dark cycle. *C. reinhardtii* was obtained from aerated semi-continuous cultures grown in modified WC medium with vitamins (Guillard 1975) under low-light conditions (~ 55 µmol photons m^−2^ s^−1^) to ensure high nutrient concentrations in the algae.

### Preparation of kairomone extract

The kairomone was extracted from frozen *Chaoborus flavicans* larvae (Vita mygglarver = ‘white mosquito larvae’, Akvarie Teknik, Sweden) according to the protocol of Tollrian (1995). In short, 100 g frozen *Chaoborus* larvae were boiled in 200 mL water for 10 min and larvae were removed afterwards using mesh gauze. Particles were removed by centrifugation (4000 rpm, 20 min) and subsequent filtration (0.1 µm, Vacuum filtration, Filtropur V50 500 mL, Sarstedt). The extract was further purified by solid-phase extraction using a C18 solid-phase cartridge (10 g of sorbent, volume 60 mL, Mega Bond Elut, Agilent Technologies). The extract was distributed to 1.5 mL tubes and stored at − 20 °C until use in experiments.

### Exposure experiments

In general, adult females of the three collected clones carrying eggs of the 3rd clutch in their brood pouch (mothers) were used in the laboratory exposure experiments. Mothers were always transferred daily to new jars containing freshly prepared food and kairomone suspensions until the release of their offspring (i.e. the juveniles). Mothers were then removed and the juveniles were kept until the second to fourth instar on *C. reinhardtii* as food ad libitum unless otherwise specified. Juveniles were scored individually for neckteeth induction using a microscope and were photographed using a computer-aided camera for later measurements of body length, body width, tail spine (spina) length, and crest height using ImageJ.

Gravid females of the two clones Pond5–16 and GINA-17 were kept individually in 50 mL jars filled with 40 mL ADaM medium and fed *C. reinhardtii* (0.5 mg carbon L^−1^). Four to eight females per clone were used in a control treatment (no kairomone exposure) and in a kairomone exposure treatment (addition of 40 µL *Chaoborus* kairomone extract to 40 mL ADaM). The released juveniles of these females were kept until their third and fourth instar for clone GINA-17 and Pond5–16, respectively. At each instar, generally three to five juveniles per mother were scored for neckteeth induction and photographed for later length measurements.

The third clone (AF-16) was used in a larger experiment, manipulating the concentration of phosphorus (P) in the food algae to assess the effect of P-limitation on neckteeth induction (Rinehart et al. unpublished). A subset of these data (P-replete conditions) is used in this study, and protocols vary to some extent from the experiments with the other clones. Gravid females of the AF-16 clone were kept in two jars filled with 1 L COMBO medium (Kilham et al. 1998, without P and N stock solutions) and fed the green algae *C. reinhardtii* (2 mg carbon L^−1^). 50 females were kept in each jar and one jar served as control treatment (no kairomone exposure) and the other jar served as the kairomone exposure treatment (addition of 750 µL *Chaoborus* kairomone extract, i.e. 30 µL per 40 mL ADaM).

The released juveniles of these females were randomly distributed to six jars per kairomone treatment with eight juveniles per jar. The jars contained 100 mL COMBO medium (without P and N stock solutions) and juveniles were kept until the third instar and fed 2 mg carbon L^−1^ per day of frozen *C. reinhardtii* (C:P = 100). The juveniles were transferred daily to new jars containing freshly prepared food suspensions. Each day, 2 randomly selected individuals per jar were scored for neckteeth induction and photographed for later length measurements (crest height could not be measured due to insufficient resolution of the pictures).

### Chaoborus kairomone gradient

To test the sensitivity of neckteeth induction to predation threat, gravid females of the clone Pond5–16 were kept individually as described in the exposure experiment above, but on a kairomone concentration gradient. Six kairomone concentrations were applied (0, 1, 2.5, 5, 10, 20 µL kairomone extract added to 40 mL ADaM, corresponding to a gradient between 0 and 0.5 µL extract mL^−1^ medium), and four females were used per concentration. The released juveniles of these females were kept until the second instar and generally three to five juveniles per mother were scored for neckteeth induction. We also used a conversion factor between the purified kairomone extract and *Chaoborus* density established by Hammill et al. (2008), where 0.5 µL extract mL^−1^ corresponds to 26 *Chaoborus* larvae L^−1^, to assess the level of *Chaoborus* predation threat required to induce neckteeth.

### Scoring of neckteeth and length measurements

**Fig. 1:**
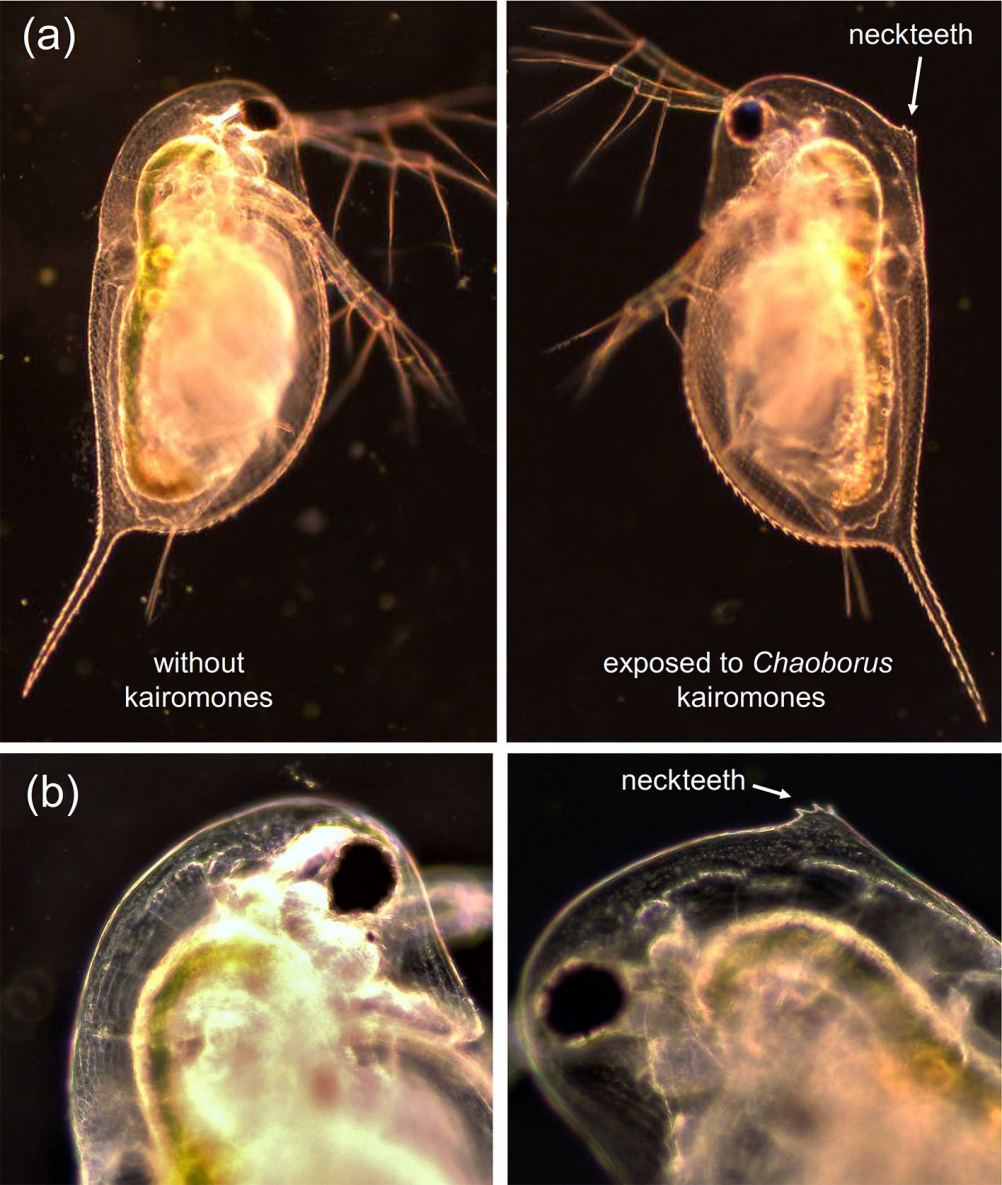
**a** Second instar *D. longispina* (Pond5–16 clone) from the control and *Chaoborus* kairomone exposed treatment. **b** Close-up of the head region of a control (left panel) and kairomone exposed (right panel) individual, the latter showing a pedestal and 3 neckteeth

Neckteeth induction of juveniles was scored according to Tollrian (1993) on live individuals using a microscope. Neckteeth (i.e. small spines at the dorsal head margin, see Fig. 1) were scored 10% each and very small teeth were scored 5%. At the base of the neckteeth, a pedestal of varying size can develop and was scored 30% when small, 50% when large, and 0% when absent. The induction score per individual is the sum of the neckteeth and pedestal scores.

Body length, body width, and tail spine (spina) length of individual juveniles were measured from photographs using ImageJ with a landmark approach (see also Appendix A1). From the set landmark points, body length was calculated as the linear distance between the top of the head and the base of the spina, body width between the ventral midpoint and dorsal midpoint, and spina length between the base and the tip of the spina (Appendix A1).

Maximum crest height was measured for the clones Pond5–16 and GINA-17 from additional photographs of higher magnification using ImageJ according to Miyakawa et al. (2013). Crest height was defined as the maximum distance between the dorsal margins of two antennal muscles and the dorsal head margin (see also Appendix A2). For *Chaoborus* kairomone exposed daphnids, the dorsal head margin corresponds to the highest point of the pedestal (excluding the teeth, Appendix A2).

### Statistical analysis

Differences in measured spina length, body width, and crest height between *Chaoborus* kairomone exposed (risk) and control (no risk) animals of each clone were analyzed within each juvenile instar using an analysis of covariance (ANCOVA) with body length as the covariate to account for size differences of animals and risk treatment as the factor. Juveniles were nested within adult female replicates to account for the nested design of multiple measured juveniles originating from a single adult female (i.e. the mother). To analyze the dependence of neckteeth induction of clone Pond5–16 on kairomone concentration (*Chaoborus* kairomone gradient experiment), we fitted a sigmoid function through the induction scores using the non-linear least-square procedure. All statistical analyses and tests of their assumptions were performed using the statistical software R, version 3.3.2 (R Core Team 2016).

## Results

### Field observations

Most permanent ponds devoid of fish, or with low fish density, contained *D. longispina* if the pH was above 5.0 and the sites were not heavily polluted. Most of these ponds contained *Chaoborus* spp., such as *C. flavicans*, *C. obscuripes*, *C. crystallinus* and *C. pallidus* (Table 1). Except for *C. pallidus*, all these *Chaoborus* species are common in Norway (Nilssen 1974). In these ponds, nearly all small-sized *D. longspina* (early instars and occasionally males) carried neckteeth (> 80% of the populations on a yearly basis). If the ponds were dominated by *C. obscuripes* (the largest *Chaoborus* spp.), a greater portion of *D. longspina* males carried neckteeth. Nearly all clear-water rock-pools with low salinity contained *D. longispina*, but no *Chaoborus* spp. In these pools, neckteeth were never observed in *D. longispina*.

**Table 1:** Summarized field observations of *Chaoborus* spp. occurrence and presence of neckteeth in *D. longispina* s. str. populations based on 810 sites of aquatic ecosystems with different characteristics in Norway, Sweden, and Denmark

Types of water bodies	Number of sites	*Chaoborus* species presence	Neckteeth occurrence
Permanent ponds	*n* = 35	*C. crystallinus, C. flavicans, C. obscuripes, C. pallidus*	Very frequent (> 80%)
Rock pools	*n* = 450	Not present	Not detected
Tarns (small, humic-rich forest lakes)—O_2_ deficient hypolimnion	*n* = 30	*C. flavicans, C. obscuripes*	Very rare (< 2%)
Medium and large lakes—O_2_ deficient hypolimnion	*n* = 85	*C. flavicans*	Not detected
Medium and large lakes—O_2_ not deficient in hypolimnion	*n* = 210	Not present	Not detected

Small, humic substance-rich forest lakes (tarns), with permanent oxygen shortage in the hypolimnion, harboured high densities of *Chaoborus* spp. (*C. obscuripes* and/or *C. flavicans*) that aggregate in the hypolimnion (Table 1). In Norway, tarns mainly contain *D. lacustris* (a close relative of *D. longispina*), but occasionally also *D. longispina*. Neckteeth on *D. lacustris* have never been observed in tarns, but they have been found occasionally on *D. longispina* in these habitats (< 2% of the investigated populations).

In all of the larger lakes, *D. longispina* did not possess neckteeth, likely reflecting the general absence of *Chaoborus* larvae due to fish predation. *C. flavicans* may be present in these systems if there is oxygen depletion in deeper waters, but is elsewhere removed by fish.

### Experiments and species delimitation

Morphological as well as genetic analyses confirmed that females of the clonal lines used to obtain juveniles for experiments belong to the species *D. longispina*. The clone AF-16 could not be analyzed genetically (see methods), but showed the same morphological characteristics as the other two clones. Nuclear DNA analysis did not provide any evidence for hybrid or backcross clones.

**Fig. 2:**
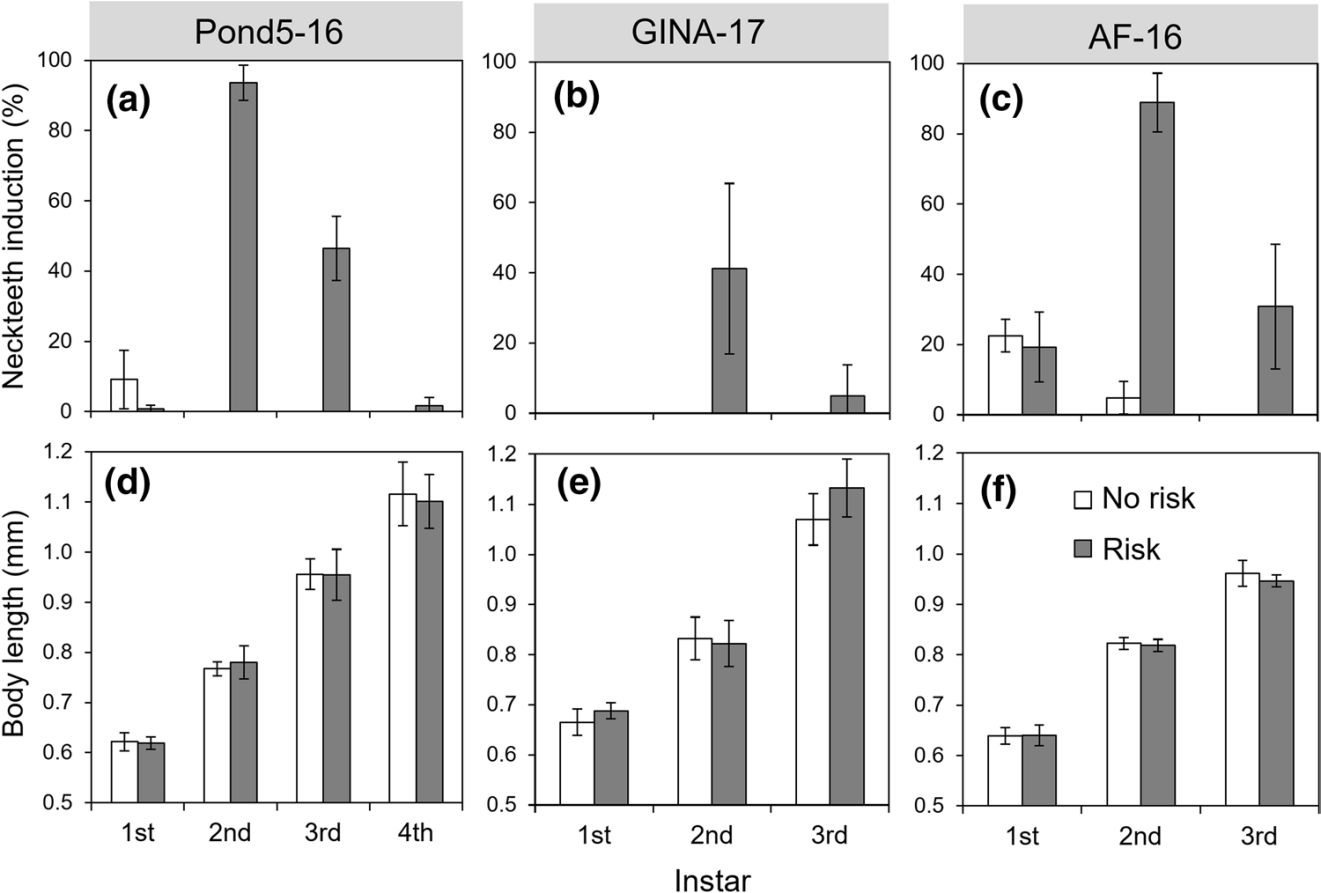
Neckteeth induction (**a**–**c**) and body length (**d**–**f**) of three *D. longispina* clones at different juvenile instars without (no risk) and with (risk) exposure to *Chaoborus* kairomones during embryonal development (mean ± SD; *N* = 4 for Pond5–16 and GINA-17, except *N* = 8 for clone GINA-17 in second instar risk treatment; *N* = 6 for AF-16, except *N* = 8 for first instar control treatment and *N* = 14 for first instar risk treatment)

Exposure of *D. longispina* to the *Chaoborus* kairomone extract during embryonal development in the brood pouch of mothers resulted in a morphotype that showed clear neckteeth expression (Fig. 1). Neckteeth induction scores were highest in the second instar, and especially high for clone Pond5–16 and AF-16 (Fig. 2). All investigated *D. longispina* clones developed up to 6 neckteeth in the second instar, but mainly 3–5 neckteeth if exposed to kairomones. The clones Pond5–16 and AF-16 showed a very similar pattern in neckteeth expression and size development over the first three instars, whereas clone GINA-17 showed lower neckteeth induction and larger growth increments between instars (Fig. 2). There was no significant difference in body length between animals exposed to *Chaoborus* kairomone and control animals (Fig. 2).

**Fig. 3:**
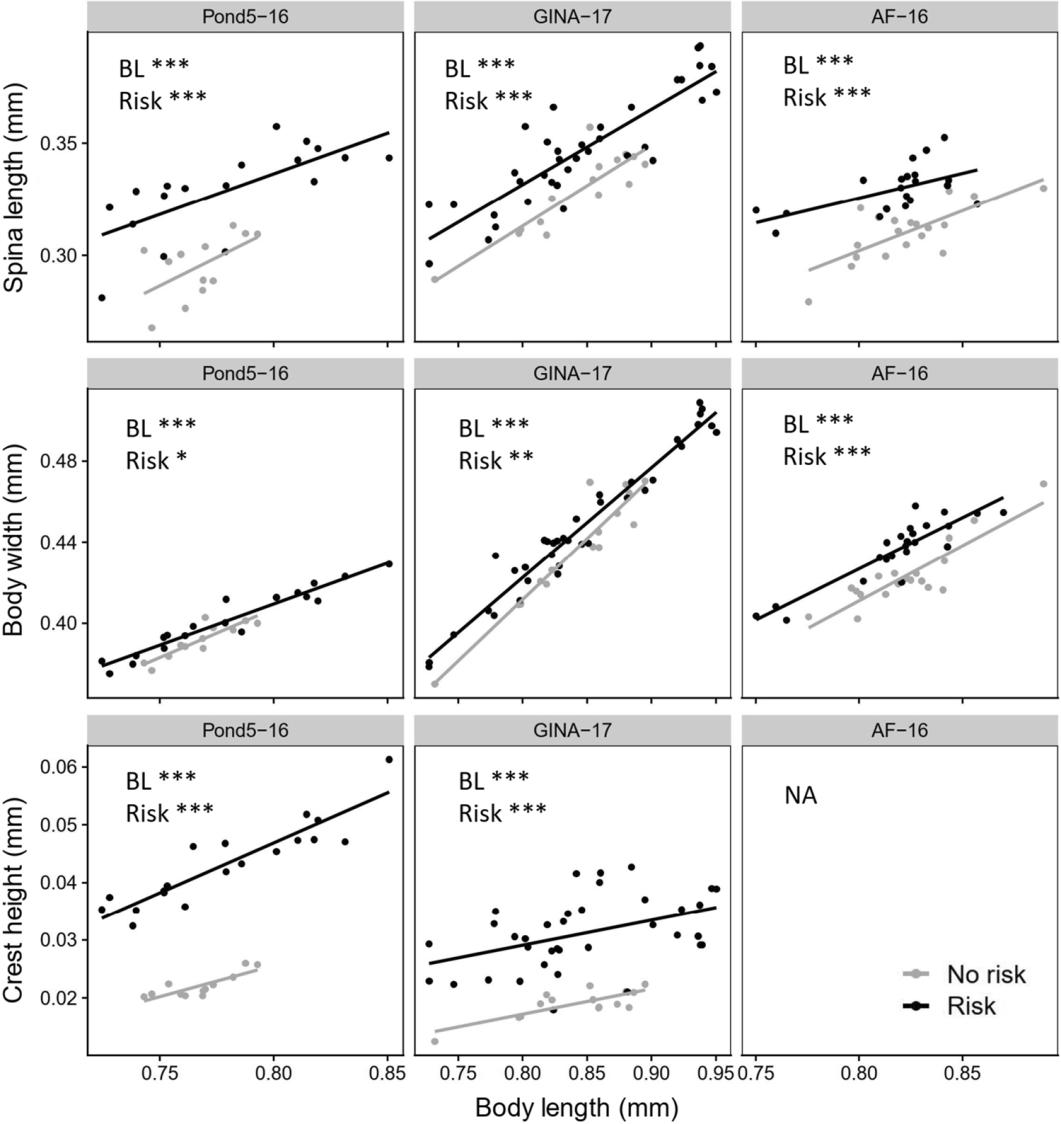
Tail spine (spina) length, body width, and crest height (depending on body length) of three *D. longispina* clones (Pond5–16, GINA-17, AF-16) at the second juvenile instar without (no risk) and with (risk) exposure to *Chaoborus* kairomone during embryonal development. Crest height could not be measured for the AF-16 clone due to insufficient image resolution. Differences between risk treatments were tested using ANCOVA with body length (BL) as covariate (*p < 0.05, **p < 0.01, ***p < 0.001)

Kairomone exposure affected spina length, body width, and crest height when accounting for differences in body length (Fig. 3). Second instar juveniles exposed to kairomone had a longer tail spine, wider body, and higher crest compared to unexposed controls (Fig. 3). These patterns were also occasionally observed in first and third instar juveniles (see supplementary material, Fig. S2, Fig. S3, Fig. S4).

**Fig. 4:**
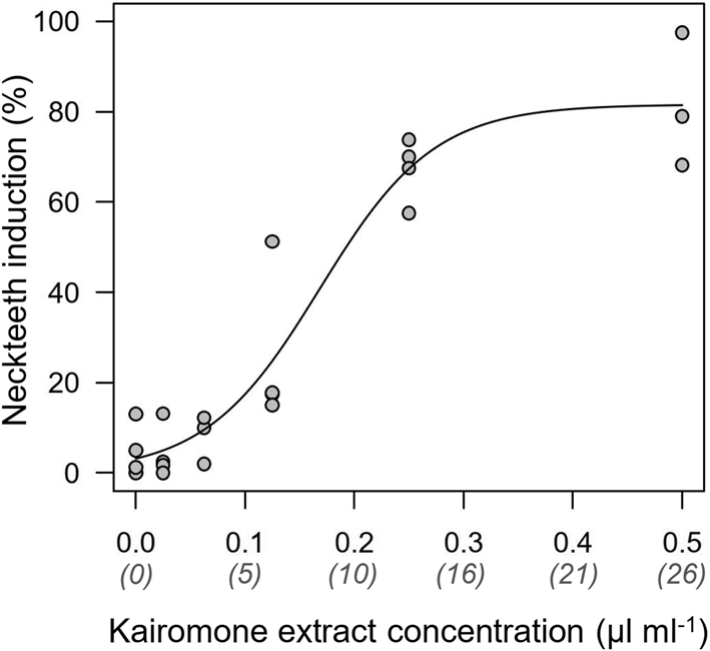
Neckteeth induction in second instar *D. longispina* (clone Pond5–16) in response to concentration of *Chaoborus* kairomone extract during embryonal development. Numbers in parentheses indicate an estimate of *Chaoborus* density based on a conversion factor between kairomone extract and *Chaoborus* density (Hammill et al. 2008). Neckteeth induction was described by a sigmoid model $= \frac{c}{{1 + a e^{ - r x} }}$, with *c* = maximum induction, *r* determining the steepness of the increase, and *a* determining the *y* intercept $\left( { = \frac{c}{1 + a}} \right)$; *c* = 81.6 ± 5.6 (estimate ± SE, *t* = 14.7, *p* < 0.001), *r* = 19.1 ± 3.6 (*t* = 5.36, *p* < 0.001), *a* = 24.8 ± 12.4 (*t* = 2.00, *p* = 0.06), *y* intercept = 3.16%

Neckteeth expression showed a sigmoid, functional response to the concentration of *Chaoborus* kairomone with a sharp increase in induction between 0.12 and 0.25 µL kairomone extract per mL medium for the clone Pond5–16 (Fig. 4).

## Discussion

### Field observations

The large survey on the occurrence of neckteeth in populations of the *D. longispina* complex across Nordic ponds and lakes revealed consistent patterns (Table 1). First, neckteeth were observed at a substantial number of sites—with most neckteeth occurrence being observed in permanent ponds where at least one *Chaoborus* species is present. Neckteeth were also occasionally found in small, brown-water lakes (tarns) where a large part of the water column is anoxic. The anoxic hypolimnion in tarns can offer a fish-free refugium for *Chaoborus* larvae. Most of the surveyed clear-water, infra-saline rock-pools contained *D. longispina*, but no *Chaoborus* spp. were observed. *Chaoborus* larvae may be rare in these ponds due to predation by large-bodied invertebrate predators, such as Dytiscidae, Odonata and predatory Corixidae/Notonectidae (Ranta 1982; Nyman et al. 1985; Hädicke et al. 2017), or due to UVR stress—which can be high in these shallow, clear-water ecosystems (Lindholm et al. 2016). Interestingly, *D. longispina* never expressed neckteeth in water bodies with Corixidae/Notonectidae as the sole invertebrate predators, suggesting that only the presence of *Chaoborus* kairomone can induce neckteeth formation in this species. However, this assumption only holds if the populations have the capability to induce neckteeth—which is likely, as the *D. longispina* clones used in our laboratory studies expressed neckteeth despite coming from sites that lacked *Chaoborus* larvae. This is especially true for the clone from the island rock pool (Pond5–16), which was likely isolated from *Chaoborus* predation for a longer time. This suggests that neckteeth development is an inherent property that is deeply anchored in the genotype, although a recent introduction of this clone from an onshore lake population cannot be excluded with certainty.

Neckteeth were likely not observed in various lakes due to fish predation—as fish diminish *Chaoborus* populations in the absence of oxygen-deficient refugia (Nilssen 1974). Additionally, different chaoborids may affect neckteeth induction differently (e.g. Riessen and Trevett-Smith 2009). For example, we observed that large *C. obscuripes* frequently led to neckteeth induction in *D. longispina*, even on a large portion of males. Predation threat by *Chaoborus* larvae is also not permanent over time, even in sites where it is present, as only certain stages of *Chaoborus* larvae feed on daphnids (mainly larval instar four, but also instar three of *C. obscuripes*).

Despite the presence of chaoborids, *D. longispina* rarely developed neckteeth in the small, humic-rich tarns. Our laboratory study showed that the clone originating from a humic-rich rock pool (Pond5–16) can exhibit strong neckteeth development, despite constant absence of *Chaoborus* larvae in this habitat. However, our dose response experiment using the same clone showed a strong reaction norm of neckteeth induction in response to kairomone concentration. Substantial neckteeth induction appears only above a certain threshold of kairomone concentration (Fig. 4), likely to avoid costs of neckteeth production when kairomone concentration (and predator density) is low (e.g. Hammill et al. 2008). Neckteeth occurrence may have been rare in humic tarns, because kairomone concentrations in these environments were below the threshold for neckteeth induction. Rising CO_2_ concentrations in freshwaters might be an alternative explanation for the absence of neckteeth, because increased *p*CO_2_ levels have been shown to suppress the formation of neckteeth and crests in daphnids (Weiss et al. 2018b). Norwegian lakes and tarns might be especially susceptible for the uptake of anthropogenically produced CO_2_ due to the rarity of limestone and the associated low buffering capacity of water bodies. Also density-dependent adjustments of inducible defenses may play a role in the tarns as it has been shown that high densities of conspecifics and congeners decrease defense expression (Tollrian et al. 2015).

Kairomone concentrations above the induction threshold of neckteeth are more likely to occur in permanent ponds, since *Chaoborus* population densities can be very high in these habitats. Permanent ponds can also harbor a greater diversity of *Chaoborus* species, creating a predator community with partially overlapping life cycles that can increase and prolong the presence of kairomones. Additionally, since ponds are generally warmer than tarns and lakes, ontogenetic (including embryonic) development of daphnids is more rapid, shortening the predation-free window for a given generation.

### Historical records

**Fig. 5:**
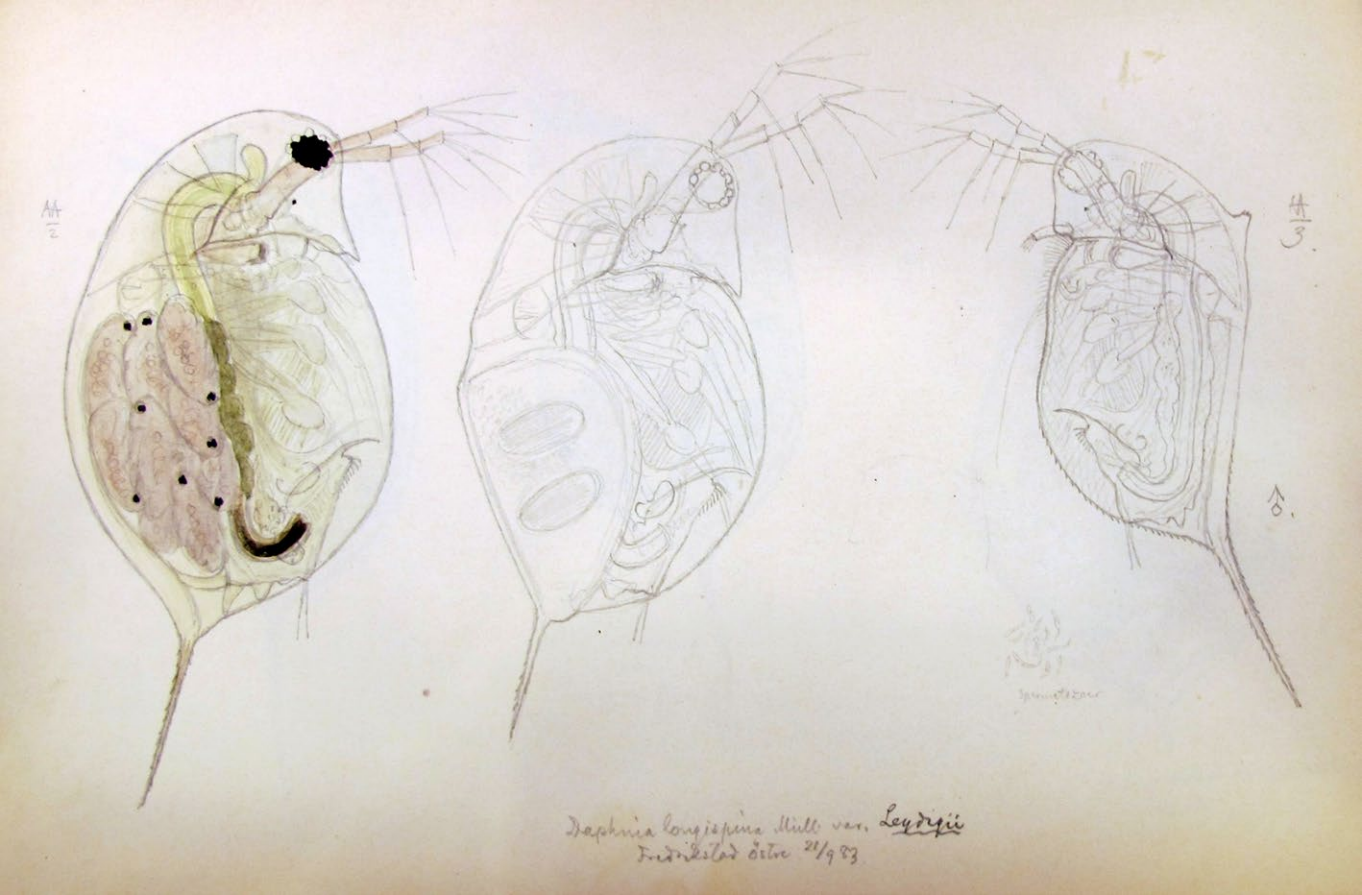
Neckteeth with strong pedestal located at the dorsal head margin of an adult male of *D. longispina*, morphotype *rosea* (specimen on the right hand side), here called *Leydigi* (Fredrikstad, Norway, 21st September 1883). This is an original, unpublished drawing by G.O. Sars (Norway: National Library Manuscript Department, Ms Fol. 1109, Item 294)

Historical records confirm that Holarctic species of the *Daphnia longispina* complex can form neckteeth in the field and under laboratory conditions, as well as in several juvenile instars both of females and males. Sars in 1883 (G. O. Sars, unpubl. data, Norway: National Library Manuscript Department, Ms Fol. 1109, Item 294: adult male of *D. rosea* morphotype, Fig. 5) and Lilljeborg (Lilljeborg 1901; Table 4, Fig. 9) observed neckteeth in *D. longispina* morphs before it was described by Brehm (1909). Berg (Berg 1931; Plate 1, Fig. 4) induced neckteeth in the *hyalina* morphotype, another main Palaearctic morphotype of *D. longispina* s.str. (Petrusek et al. 2008). The sister species of *D. longispina* in North America [presently named *D. dentifera* (Brooks 1953)] also develops neckteeth, as demonstrated in the original collection by S. A. Forbes from 1890 (Brooks 1953: Fig. 3c, female instar 3, Fig. 3g, adult male). Thus, the entire *D. longispina*-*dentifera* species complex, distributed over the whole Holarctic region, can develop neckteeth in nature (Benzie 2005; Juračka et al. 2011). The historical drawing from Sars (Fig. 5) also points to a hitherto neglected aspect in research by showing neckteeth (and the associated pedestal) formation on an adult male individual. Previous research on neckteeth induction has primarily focused on juvenile females, and juvenile males may have been investigated by chance or perhaps remained undiscovered. However, the drawing shows that natural selection has favoured neckteeth development also in males, even in adult specimens probably due to their smaller size compared to adult females.

### Laboratory exposure experiments

All three clones tested in the laboratory showed the strongest neckteeth induction scores during instar two, but clones differed in the magnitude of neckteeth induction during instars one and three. This corresponds to our in situ observations, where population-level variation in neckteeth expression may result from different combinations of *Chaoborus* species, *Chaoborus* densities, or *Daphnia* clone-specific properties. Allometric responses in the experiments also differed somewhat across clones. Specifically, clones varied in regard to the slope of the response with size and the difference between exposed and control animals. However, for all clones, body width was less responsive than spina length and crest height. Clones from eastern Norway (Pond5–16 and AF-16) showed similar, strong neckteeth expression, despite originating from ponds without *Chaoborus* larvae. Parejko and Dodson (1991) also induced neckteeth expression in *D. pulex* clones originating from *Chaoborus*-free ponds in the laboratory, although clones from predator-free ponds generally showed weaker responses than clones from ponds containing the predator.

The expression of inducible defense traits observed for *D. longispina* is consistent with studies on *D. pulex*. Neckteeth in *D. longispina* were induced during early instars, when juveniles were small. In fact, *D. longispina* showed the strongest expression in instar two, a pattern often also observed for *D. pulex* (e.g. Tollrian 1993, 1995). The morphology of the neckteeth, and the concomitant protrusion at their base (i.e. the ‘pedestal’), observed on our *D. longispina* clones was very similar in appearance to those expressed by *D. pulex* (e.g., Tollrian 1993; see also Appendix 3). For example, the crest height (a measure dependent on the presence of the pedestal) was larger in the induced phenotype than in the non-induced phenotype of the investigated *D. longispina* clones, a pattern previously observed also in *D. pulex* (Imai et al. 2009; Miyakawa et al. 2013). Additionally, in agreement with previous studies on *D. pulex* (e.g. Lüning 1992; Imai et al. 2009; and references in Riessen and Gilbert 2019), we observed elongated tail spines in response to kairomones in all three *D. longispina* clones. A longer tail spine may make juvenile daphnids more difficult to handle for their gap-limited *Chaoborus* predator. We also observed increased body width in *D. longispina* exposed to kairomones, which may also protect daphnids from gape-limited *Chaoborus* larvae (Riessen and Trevett-Smith 2009).

Neckteeth expression was also dependent on *Chaoborus* kairomone concentration for one of our investigated *D. longispina* clones. Such concentration-dependent responses have been previously observed in numerous *D. pulex* clones (Havel 1985; Parejko and Dodson 1991; Tollrian 1993; Hammill et al. 2008; Dennis et al. 2011; Carter et al. 2017). The sensitivity and magnitude of these responses appear to depend on the historical predation regime of the habitat. For instance, *D. pulex* clones from *Chaoborus*-free ponds generally showed weaker responses to imposed predation threat than clones from *Chaoborus* containing ponds (Parejko and Dodson 1991). Similarly, clones from *Chaoborus*-harboring but fishless ponds showed higher neckteeth induction at lower kairomone concentrations than clones from ponds with *Chaoborus* and fish (Hammill et al. 2008; Dennis et al. 2011; Carter et al. 2017). Thus, the occurrence of neckteeth along the gradient of predation threat may depend on the duration a particular clone has spent in a predator-scarce environment in combination with the strength of selection against neckteeth formation (assuming fish can control *Chaoborus* densities to low levels). Our tested *D. longispina* clone showed a sensitive and strong response to the predator cue that was similar to the responses of *D. pulex* clones from *Chaoborus*-harboring but fishless ponds (even though our clone originated from a *Chaoborus*-free rock pool). Similar to the *D. pulex* clones from the *Chaoborus*-ponds (cf. Hammill et al. 2008; Dennis et al. 2011; Carter et al. 2017) our clone showed close to maximum induction (70–80%) at 0.25 µL extract mL^−1^, which corresponds to a *Chaoborus* density of ~ 13 larvae L^−1^ (Hammill et al. 2008). This density may seem a little high compared to natural densities, but note that neckteeth can be induced already at densities < 13 larvae L^−1^ (Fig. 4; Hammill et al. 2008).

Despite recent progress in investigating the *Chaoborus* capture process, the mechanism of the predator-defensive role of the neckteeth is still not fully understood (Weiss et al. 2018a; Kruppert et al. 2019), and although being inducible, the costs for developing neckteeth may be modest (Tollrian and Dodson 1999). However, neckteeth development is often accompanied by other, seemingly more costly, traits such as an elongated tail spine, increased pedestal size, and increased carapace thickness, strength, or stiffness (Laforsch et al. 2004; Riessen et al. 2012; Rabus et al. 2013; Kruppert et al. 2017; Riessen and Gilbert 2019). Nevertheless, the similarity in neckteeth and pedestal expression in *D. pulex* and *D. longispina* as observed in this study (Appendix 3) suggests that these induced morphological defense traits are evolutionarily very efficient in protecting against predation from *Chaoborus* larvae.

### Evolutionary aspects

Revealing whether these phenotypic differences are of genotypic or epigenetic origin may require extensive genomic or transcriptomic profiling and comparisons across clones. Comparative transcriptional profiling of individuals with and without induced traits would be required to determine the evolutionary origin and relatedness of these traits in various species or species complexes. This may not be straightforward, since a large number of genes are involved (Miyakawa et al. 2015; Christjani et al. 2016; Hales et al. 2017; An et al. 2018). In *D. pulex*, for instance, different expression patterns were reported in 230 genes (158 up- and 72 downregulated) in response to predator-induced neckteeth development (Rozenberg et al. 2015). Among these were genes related to structural functions (cuticle genes), as well as genes related to metabolic processes. Hormonal pathways are also likely involved (Dennis et al. 2014; Weiss et al. 2015). For example, dopamine signaling has been found to be strongly associated with predator-induced defenses in *D. pulex* and *D. longicephala* (Weiss et al. 2015). A further screening of this will likely narrow down candidate genes, but still a comparative study across species and haplotypes will be a long endeavor.

Our study confirms that induced morphological defenses in response to *Chaoborus* kairomones occur in the *D. longispina* complex. These data also provide insights into the types of sites and conditions that promote these responses, which are similar to the shape observed in the *D. pulex* complex (see Appendix 3). The similarity in phenotypic plasticity across these two distantly related species complexes is puzzling, as it clearly differs from responses in species such as *D. cucullata, D. cristata* and to some extent *D. galeata*, which produce different types of helmets or crests, but never neckteeth (Beaton and Hebert 1997; Riessen and Gilbert 2019). The similar shape of neckteeth formation in the two distantly related groups may suggest a shared, rather than an independent evolutionary origin of this trait (Juračka et al. 2011). Following this line, the ancestral trait might have been lost in more ‘modern’ species without strong selection pressure by *Chaoborus* predation.

However, the presence of neckteeth in the *D. pulex* as well as *D. longispina* complex does not necessarily imply a single ancestral origin. The formation of various head shapes in different *Daphnia* species (i.e. helmets, crests, neckteeth) is determined by the number and location of polyploid cells in the cephalic epidermis, which regulate accelerated cell division of surrounding diploid cells (Beaton and Hebert 1997). The presence of polyploid cells might be a shared, ancestral trait in daphnids, but their number and location may be altered in different lineages to produce various changes in head shape. As with the development of similar helmet shapes in different *Daphnia* lineages, neckteeth could have evolved independently in the *D. pulex* and *D. longispina* complex in response to selection pressure by predation of *Chaoborus* larvae. Thus, further studies are required to resolve the question about the evolutionary origin of neckteeth formation.

## Electronic supplementary material

Below is the link to the electronic supplementary material.

Supplementary file1 (PDF 1117 kb)
