# Supplementary material for: Ecology of predator-induced morphological defense traits in Daphnia longispina (Cladocera, Arthropoda)
Source: Oecologia. 2020 Jan 16;192(3):687–98. doi: 10.1007/s00442-019-04588-6 (PMC7058565; doi:10.1007/s00442-019-04588-6)
Supplement: Supplementary file 1 — Supplementary file1 (PDF 1117 kb) [file 442_2019_4588_MOESM1_ESM.pdf]

Ecology of predator-induced morphological defense traits in *Daphnia longispina* (Cladocera, Arthropoda)

Erik Sperfeld[1,2,*], Jens Petter Nilssen[3], Shelby Rinehart[4,5], Klaus Schwenk[6], Dag Olav Hessen[4]

1 Animal Ecology, Zoological Institute and Museum, University of Greifswald, Greifswald, Germany

2 Centre for Ecological and Evolutionary Synthesis (CEES), Department of Biosciences, University of Oslo, Blindern, Oslo, Norway

3 Müller-Sars Society for Free Basic Research, P.O. Box 5831, N-0308 Oslo, Norway

4 Section for Aquatic Biology and Toxicology (AQUA), Department of Biosciences, University of Oslo, Blindern, Oslo, Norway

5 Department of Ecology, Evolution, and Behavior, The Hebrew University of Jerusalem, Jerusalem, Israel

6 Molecular Ecology, Institute for Environmental Sciences, University Koblenz-Landau, Landau in der Pfalz, Germany.

* corresponding author: eriksperfeld@googlemail.com

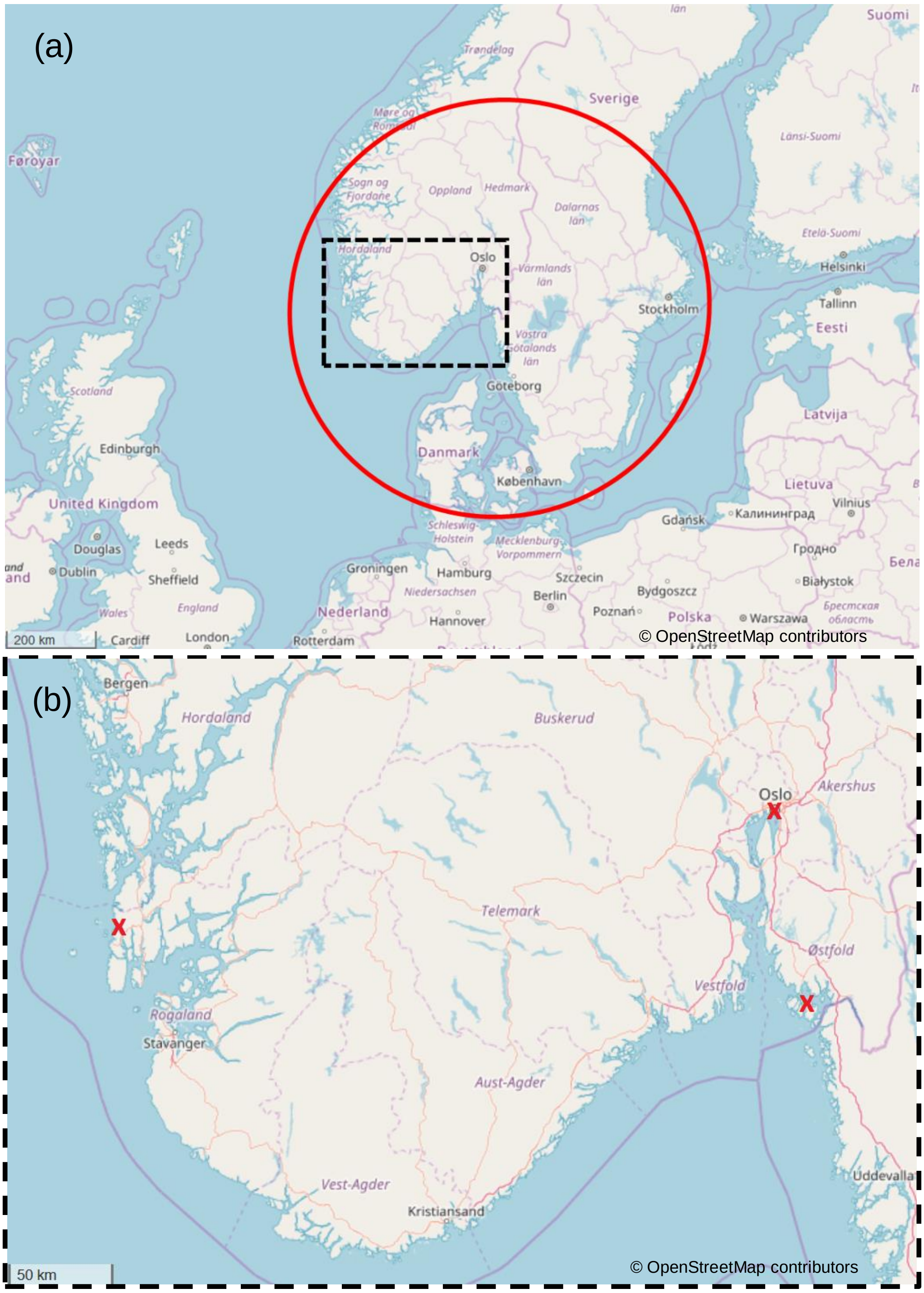

Fig. S1 (a) Main geographical region of sampled habitats (red circle). Outside this main sampling area, only around 100 lakes, tarns, or ponds have been sampled in North Norway, North Sweden, Finland, Estonia and Lithuania. (b) Sampling locations of the three clones investigated in the laboratory experiments is indicated with a red "**x**".

Appendix A1: Measuring body length, body width, and tail spine (spina) length

Body length, body width, and tail spine (spina) length of individual juveniles were measured from photographs using ImageJ with a landmark approach. A line was drawn from the base of the spina to the end of the head by passing through the middle of the eye (Fig. A1). To this anterior-posterior axis, a dorsoventral axis line was drawn automatically with an angle of 90°. Five landmarks were set: 1) top of the head (point where anterior-posterior axis line ends at the head), 2) point at ventral side of the dorsoventral axis line, 3) point at the base of the tail spine where maximum curvature is observed (spina base), 4) point at tip of spina (spina end), and 5) point at dorsal side of the dorsoventral axis line (dorsal midpoint). Body length was calculated as the linear distance between 1 and 3, body width between 2 and 5, and spina length between 3 and 4.

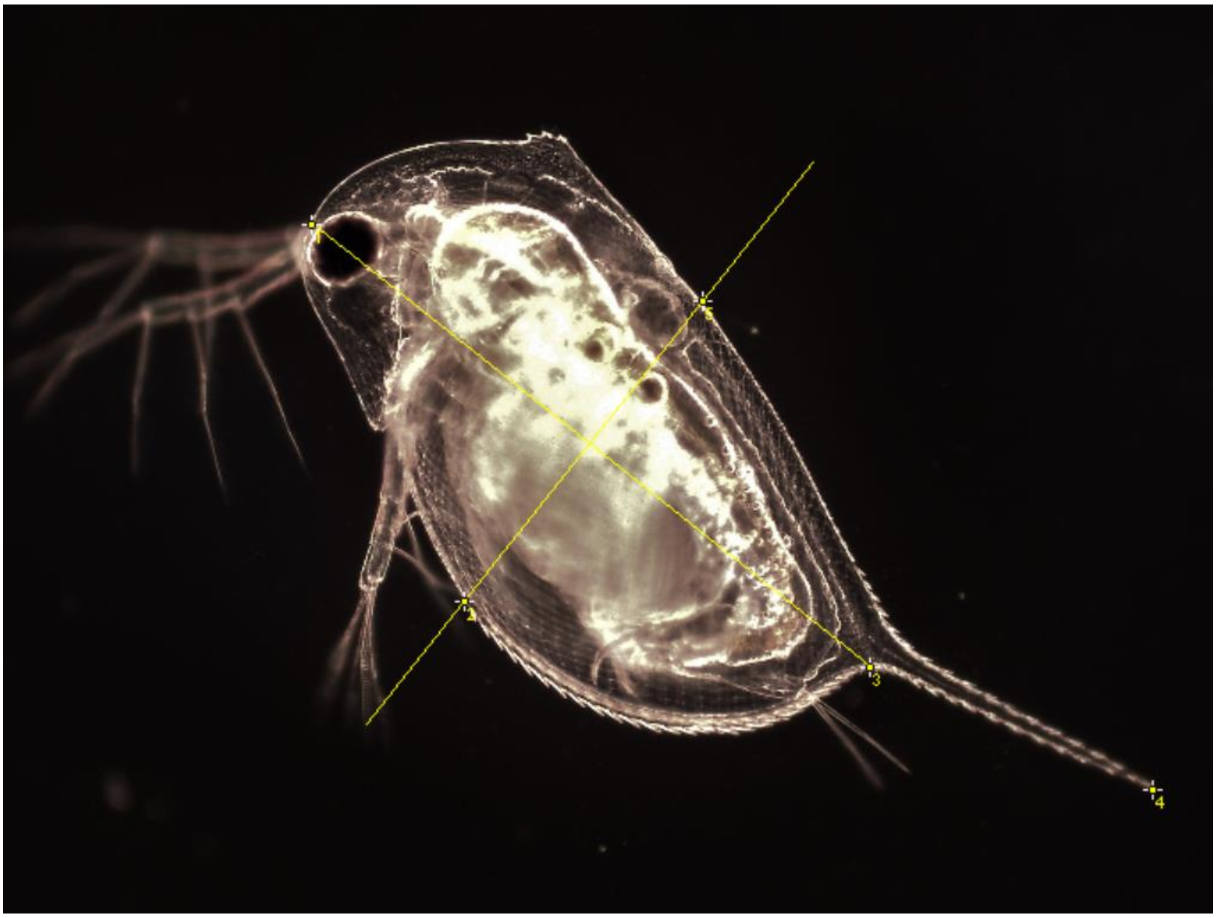

Fig. A1 Second instar juvenile of *D. longispina* clone Pond5-16 (exposed to *Chaoborus* kairomone).

Appendix A2: Measuring crest height

Maximum crest height was measured from additional photographs of higher magnification using ImageJ. A line was drawn along the anterior-posterior axis that passed antennal muscle 1 and 2 as a tangent on their dorsal margins (Fig. A2). An additional dorsoventral axis line was drawn rectangular to this anterior-posterior axis line in a way that maximized the distance between the anterior-posterior axis line and the dorsal head margin, which was defined as crest height. For *Chaoborus* kairomone exposed daphnids, crest height was the distance between the anterior-posterior axis line and the highest point of the pedestal (excluding the teeth).

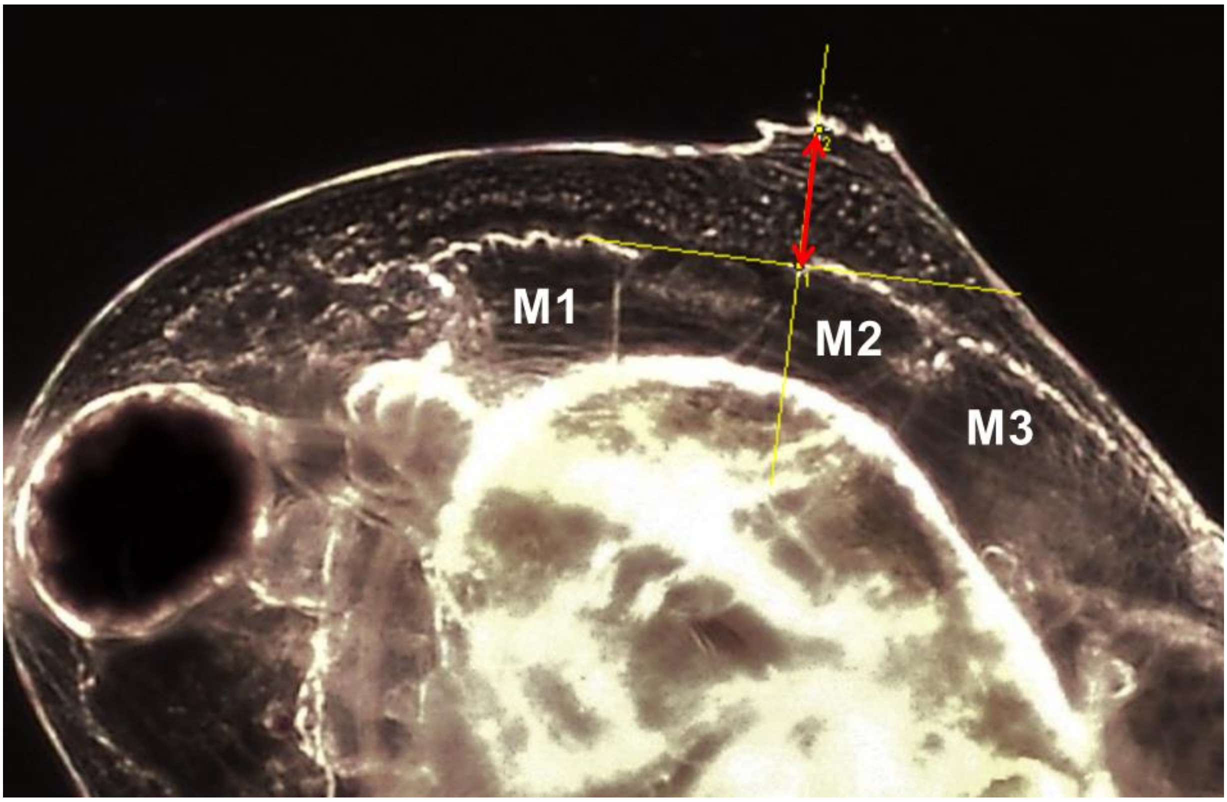

Fig. A2 Second instar juvenile of *D. longispina* clone Pond5-16 (exposed to *Chaoborus* kairomone); M1, M2, M3 are antennal muscles number 1, 2, 3, respectively.

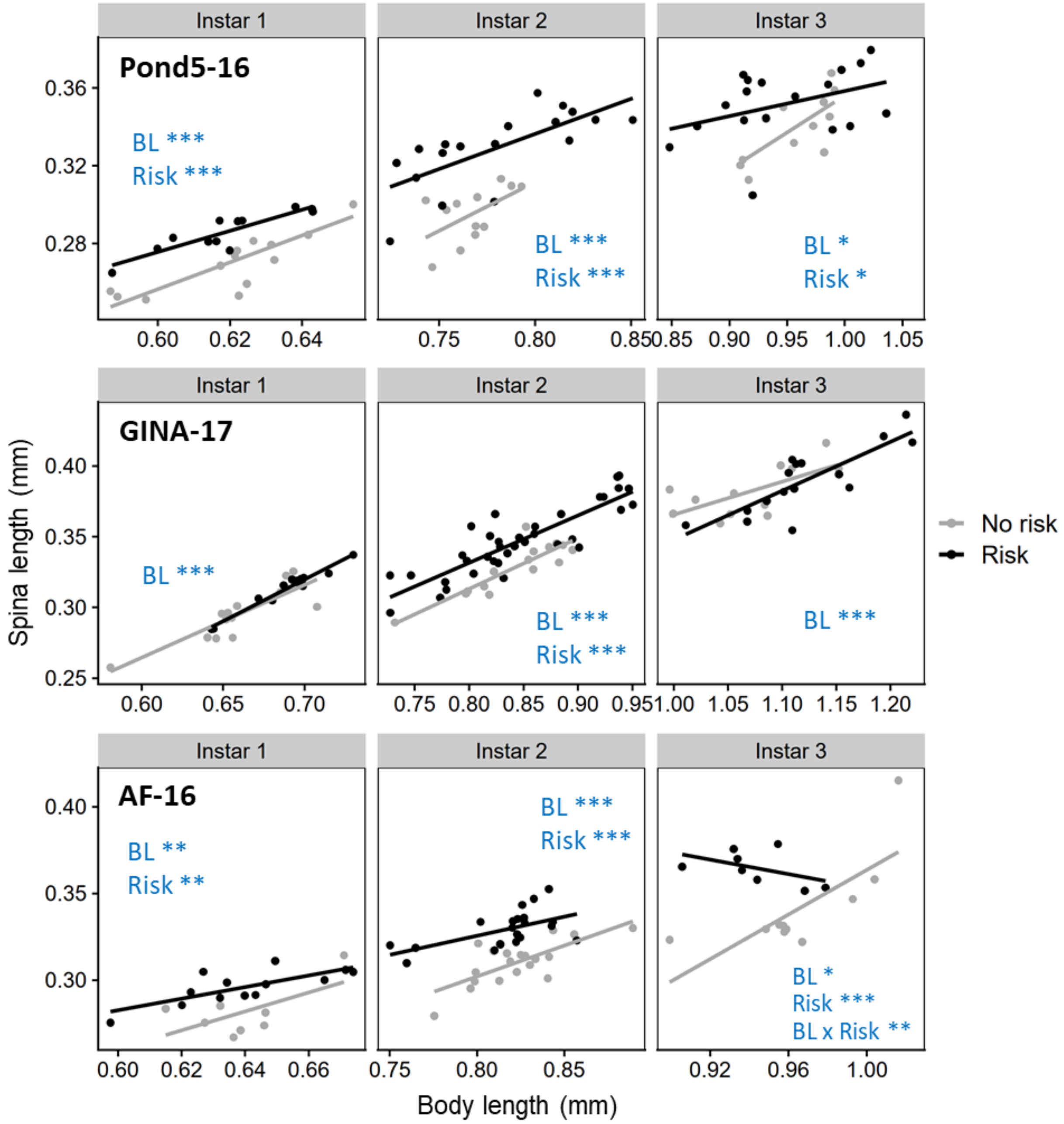

Fig. S2 Tail spine (spina) length depending on body length of three *Daphnia longispina* clones (Pond5-16, GINA-17, AF-16) at different juvenile instars without (no risk) and with exposure (risk) to *Chaoborus* kairomone during embryonal development. Differences between risk treatments were tested using ANCOVA with body length (BL) as covariate (* p < 0.05, ** p < 0.01, *** p < 0.001).

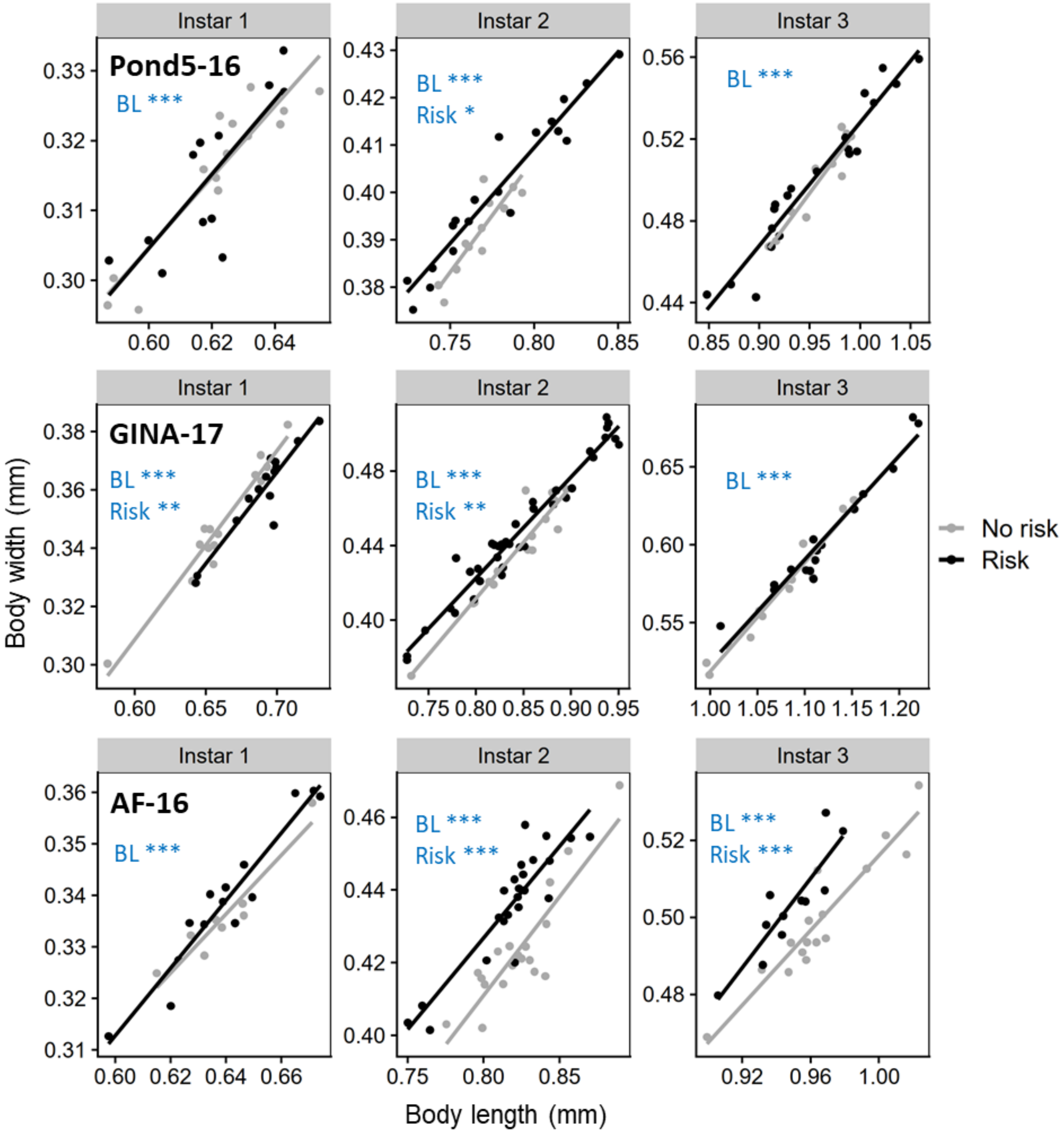

Fig. S3 Body width depending on body length of three *Daphnia longispina* clones (Pond5-16, GINA-17, AF-16) at different juvenile instars without (no risk) and with exposure (risk) to *Chaoborus* kairomone during embryonal development. Differences between risk treatments were tested using ANCOVA with body length (BL) as covariate (* $p < 0.05$, ** $p < 0.01$, *** $p < 0.001$).

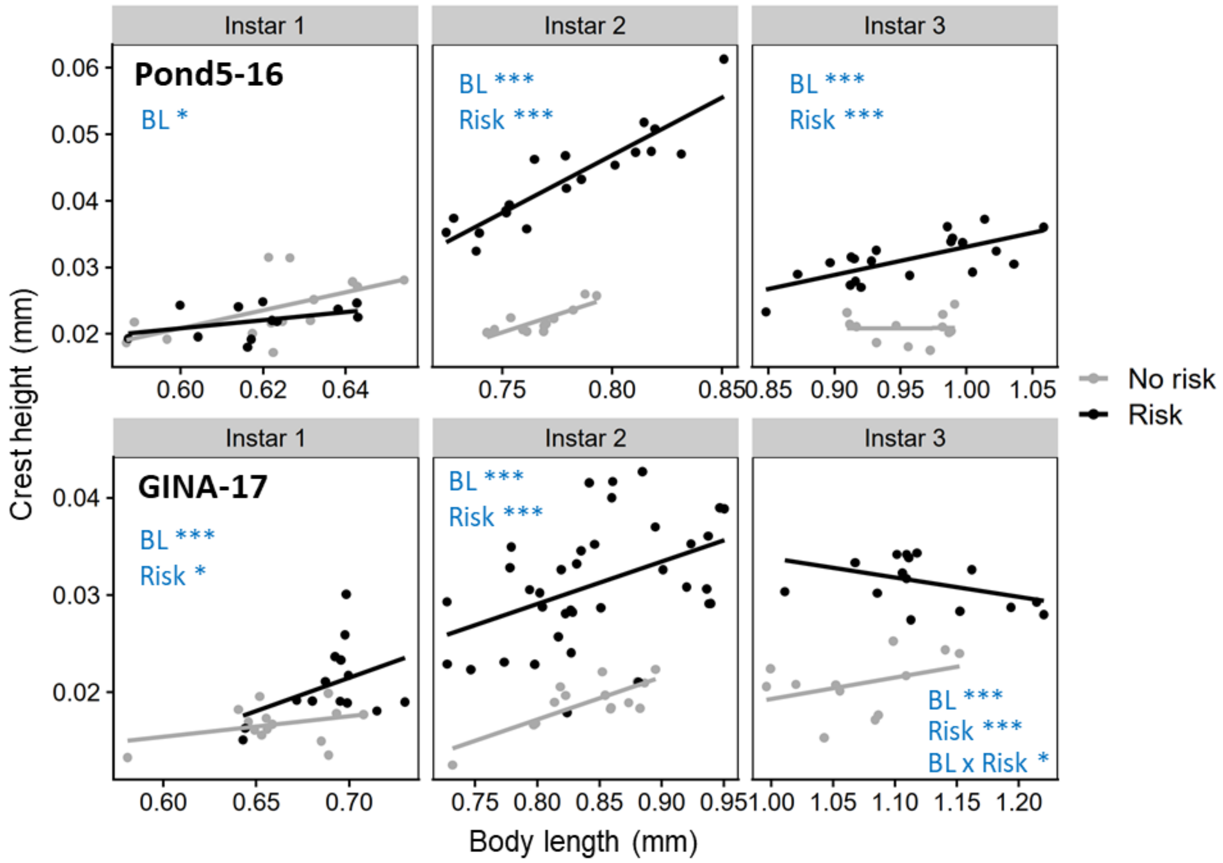

Fig. S4 Crest height depending on body length of two *Daphnia longispina* clones (Pond5-16, GINA-17) at different juvenile instars without (no risk) and with exposure (risk) to *Chaoborus* kairomone during embryonal development. Crest height could not be measured for the AF-16 clone due to insufficient resolution of the pictures. Differences between risk treatments were tested using ANCOVA with body length (BL) as covariate (* $p < 0.05$, ** $p < 0.01$, *** $p < 0.001$).

Appendix A3: Comparison of *D. longispina* and *D. pulex* morphotypes in their first two instars

Juveniles of *D. longispina* and *D. pulex* have a generally different habitus; *D. longispina* appears to have a much longer tail spine in contrast to *D. pulex*, which seems to have a bulkier body.

	**Without exposure to *Chaoborus* kairomones**	**With exposure to *Chaoborus* kairomones**
D. longisp. **Instar 1**	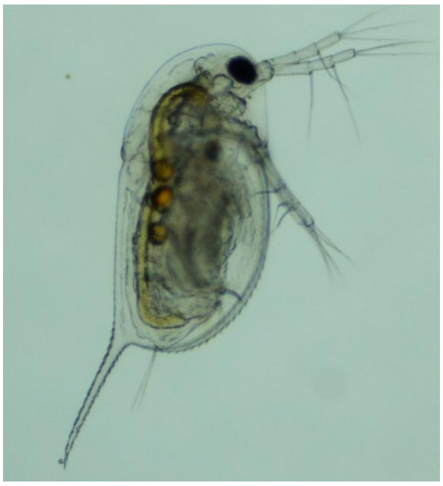 (clone Pond5-16)	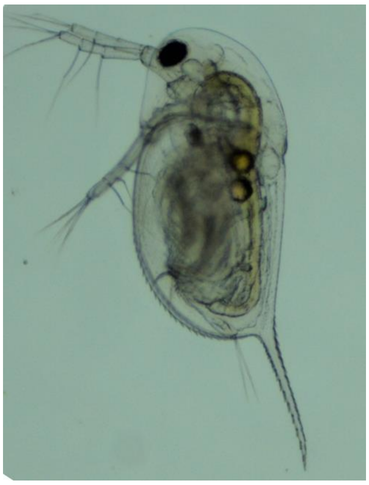 (clone Pond5-16)
D. pulex **Instar 1**	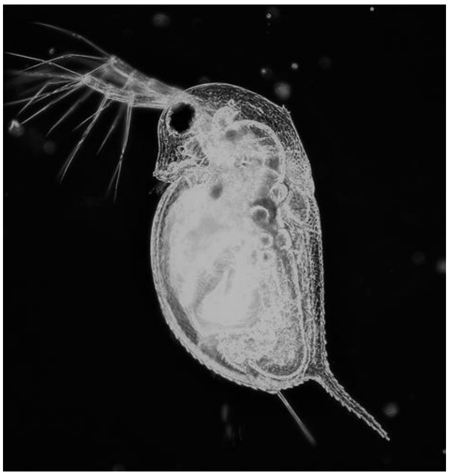 (clone SD-V-16)	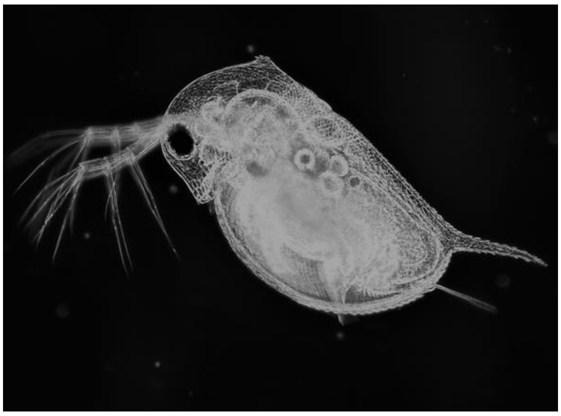 (clone SD-V-16)

Appendix A3: Continued

	Without exposure to *Chaoborus* kairomones	With exposure to *Chaoborus* kairomones
D. longisp. **Instar 2**	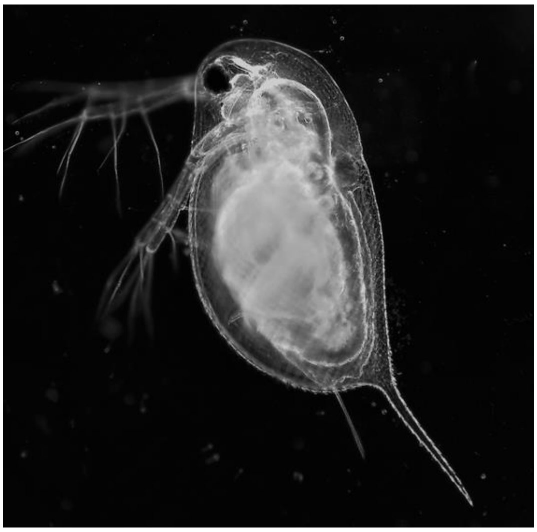 (clone Pond5-16)	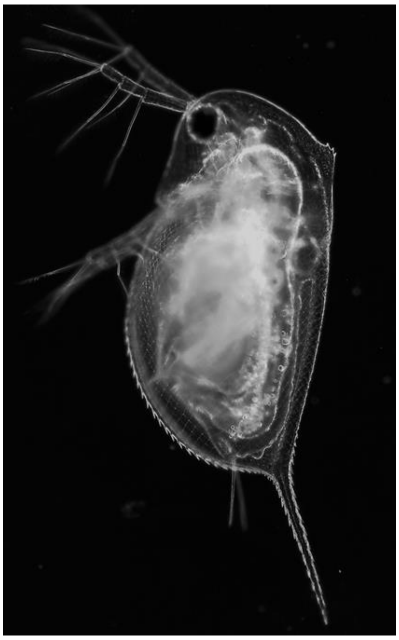 (clone Pond5-16)
D. pulex **Instar 2**	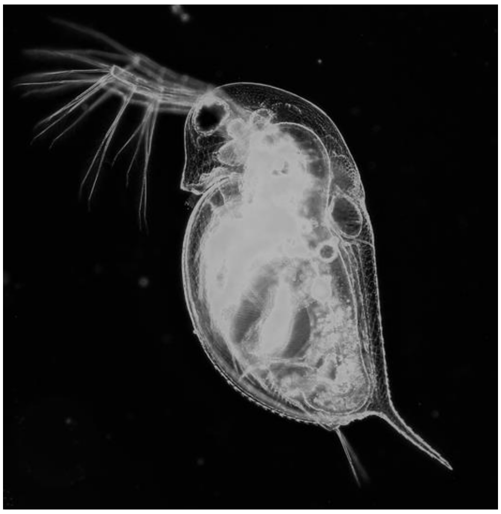 (clone SD-V-16)	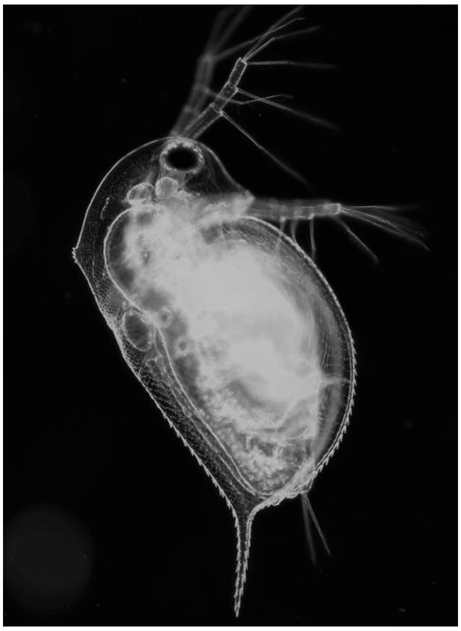 (clone SD-V-16)

Footnote: The *D. pulex* clone (SD-V-16) was collected on 27th June 2016 in an almost dried out rock pool (1x2m, depth ~20cm) that contained brownish clear water (GPS: 59.950764, 10.836059). *Chaoborus* sp. larvae were present at high density at the time of sampling. The *D. pulex* clone was maintained and treated in the same way as the *D. longispina* clones.
